# Factors affecting intradiscal pressure measurement during *in vitro* biomechanical tests

**DOI:** 10.1186/1748-7161-10-S2-S1

**Published:** 2015-02-11

**Authors:** Jaëlle Tremblay, Vladimir Brailovski, Jean-Marc Mac-Thiong, Yvan Petit

**Affiliations:** 1 Department of Mechanical Engineering, École de technologie supérieure, Montreal, H3C 1K3, Canada; 2 Research center, Hôpital du Sacré-Cœur, Montreal, H4J 1C5, Canada

## Abstract

**Objectives:**

To assess the reliability of intradiscal pressure measurement during *in vitro* biomechanical testing. In particular, the variability of measurements will be assessed for repeated measures by considering the effect of specimens and of freezing/thawing cycles.

**Methods:**

Thirty-six functional units from 8 porcine spines (S1: T7-T8, S2: T9-T10, S3: T12-T11, S4: T14-T13, S5: L1-L2 and S6: L3-L4) have been used. The intervertebral discs were measured to obtain the frontal and sagittal dimensions. These measurements helped locate the center of the disc where a modified catheter was positioned. A fiber optic pressure sensor (measuring range: -0.1 to 17 bar) (360HP, SAMBA Sensors, Sweden) was then inserted into the catheter. The specimens were divided into 3 groups: 1) fresh (F), 2) after one freeze/thaw cycle (C1) and 3) after 2 freeze/thaw cycles (C2). These groups were divided in two, depending on whether specimens were subjected to 400 N axial loading or not. Ten measurements (insertion of the sensor for a period of one minute, then removal) were taken for each case. Statistical analyses evaluated the influence of porcine specimen and the vertebral level using a MANOVA. The effect of repeated measurements was evaluated with ANOVA. The difference between freeze/thaw cycles were analysed with U Mann-Whitney test (P≤0.05).

**Results:**

Without axial loading, the F group showed 365 mbar intradiscal pressure, 473 mbar for the C1 group, and 391 mbar for the C2 group. With 400N axial load, the F group showed intradiscal pressure of 10610 mbar, the C1 group 10132 mbar, the C2 group 12074 mbar. The statistical analysis shows a significant influence of the porcine specimen (p<0.001), with or without axial loading and of the vertebral level with (p=0.048) and without load (p<0.001). The results were also significantly different between the freeze/thaw cycles, with (p<0.001) and without load (p=0.033). Repeated measurement (without load p = 0.82 and with p = 0.56) did not show significant influence.

**Conclusions:**

The results tend to support that freezing/thawing cycles can affect intradiscal pressure measurement with significant inter-specimen variability. The use of the same specimen as its own control during in vitro biomechanical testing could be recommended.

## Introduction

Biomechanical performance of spinal implants is commonly evaluated through *in vitro* tests on cadaveric spine specimens (human or animal). In order to establish the physiological loads in the spine during those tests, intradiscal pressure (IDP) could be measured. IDP can provide an overview of the load distribution at different levels of the spinal segment, intact and after instrumentation. Nachemson [1] demonstrated that the nucleus pulposus is comparable to a homogeneous fluid environment that has a hydrostatic behavior. Biomechanical measurements on cadaveric specimens, however, may be affected by different factors inherent to *in vitro* testing: freeze/thaw cycles, repeated test or installation of measuring equipment. Previous studies suggested that a single freeze/thaw cycle doesn’t significantly alter the biomechanical properties of bone [2], ligament [3] and muscle [4], but repeated *in vitro* biomechanical tests commonly involve various number of freezing/thawing cycles performed along several days. Tan and Uppuganti [5] found that the flexibility of the human cadaveric lumbosacral motion segments between test days was significantly affected after repeated freeze-thaw and cumulative testing cycles. Beyond the flexibility, the question remained for IDP measurement during a complete *in vitro* biomechanical testing protocol. The main objective of this study is to assess the reliability of intradiscal pressure measurement during in vitro biomechanical testing. In particular, the variability of measurements for repeated tests will be assessed by considering the effect of specimens and freezing/thawing cycles.

## Methods

Thirty-six functional units from 8 porcine spines (S1: T7-T8, S2: T9-T10, S3: T12-T11, S4: T14-T13, S5: L1-L2 and S6: L3-L4) have been used. The intervertebral discs frontal and sagittal dimensions were measured to help locate the center of the disc.

A catheter, modified with a glue mark limiting its insertion depth, was positioned at the center of the disc to ensure accurate insertion to the center of the disc. The catheter was maintained in place during all the tests. A fiber optic pressure sensor (measuring range: -0.1 to 17 bar) (360HP, SAMBA Sensors, Sweden) was inserted until the end of the catheter. The specimens were divided into 3 groups: 1) fresh (F), 2) after one cycle of freeze/thaw (C1) and 3) after 2 cycles of freeze/thaw (C2). These groups were divided in two, depending on whether specimens were subjected to 400 N axial loading or not. Ten measurements (insertion of the sensor in the catheter for a period of one minute, then removal) were taken for each case (specimen and freeze/thaw condition). Statistical analysis evaluates the influence of porcine specimens and vertebral levels using a MANOVA. The variability between the measurements was evaluated with a repeated measure ANOVA. The difference between freeze/thaw cycles were analysed with U Mann-Whitney tests (P ≤ 0.05).

## Results

**Figure 1:**
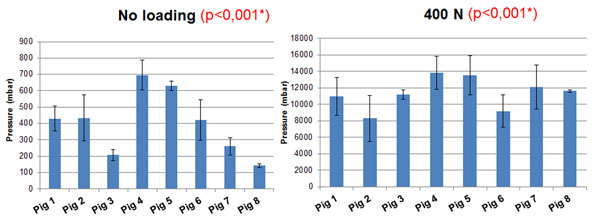
Mean pressure measurement versus porcine specimen

**Figure 2:**
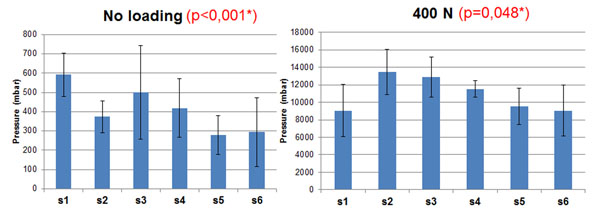
Mean pressure measurement versus vertebral level

**Figure 3:**
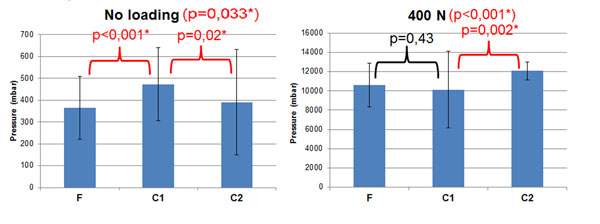
Mean pressure measurement versus number of freezing/thawing cycles

**Figure 4:**
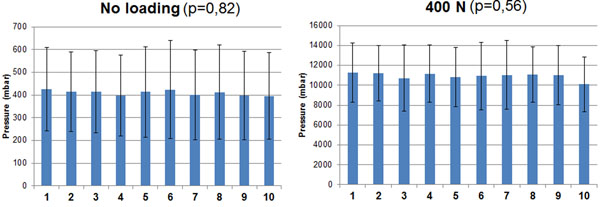
Mean pressure measurement versus number of repetitions

Without axial loading, the F group showed 365 mbar of intradiscal pressure , 473 mbar for the C1 group, and 391 mbar for the C2 group. With 400N axial load, the F group showed intradiscal pressure of 10610 mbar, the C1 group 10132 mbar, and the C2 group 12074 mbar. Figure 1 shows a significant inter-specimen variability (p<0.001) with and without axial loading, Figure 2 also shows significantly vertebral level dependent results, with (p=0.048) and without load (p<0.001). The results were also significantly different between the freeze/thaw cycles (Figure 3) with (p<0.001) and without loading (p=0.033). Repeated measurements (Figure 4) did not show significant variability (without p = 0.82 and with load p = 0.56).

## Discussion

The purpose of this study was to assess the reliability of IDP measurement during *in vitro* biomechanical testing. In particular, the variability of the measurements on repeated tests was assessed by considering the effect of specimens and of 2 freezing/thawing cycles.

The results are in good agreement with the literature [6]. The amplitude of IDP was considered normal for the specimens and the test applied.

Measured IDP varied depending on the porcine specimen and the vertebral level. It is important to consider these findings in the planning of *in vitro* biomechanical studies with IDP measurement. The interspecimen variability will make difficult to obtain significant results without using large number of specimens or using each single specimen as its own control, in paired testing comparing two conditions.

Repeated measurements (up to 10) did not significantly affect intradiscal pressure in this study. It allows designing *in vitro* biomechanical studies with multiple measurements on a single specimen at different configurations without affecting significantly the result.

Only one freezing/thawing cycle may affect the intradiscal pressure. Since IDP is based on the hydrostatic behavior of the nucleus, freezing may significantly affect this measurement. This suggests comparing results within the same freeze/thaw cycle.

## Conclusions

This biomechanical study investigates different factors that may affect IDP measurement. We recommend planning the test protocol as to avoid comparisons between specimens and freeze/thaw cycles.

This is the extended abstract of IRSSD 2014 program book [7].

## Competing interests

The authors have no conflicts of interest.

## Authors' contributions

JT carried out the IDP measurement experiments and drafted the manuscript. VB and JMMT drafted the study design and add an intellectual content to the manuscript. YP have been involved in drafting the manuscript and revising it critically for important intellectual content. All authors read and approved the final manuscript.

## Declarations

Publication charges for this collection were funded by IRSSD 2014 Sapporo.

This article has been published as part of *Scoliosis* Volume 10 Supplement 2, 2015: Research into Spinal Deformities 9: Short Papers from the IRSSD 2014 Meeting. The full contents of the supplement are available online at http://www.scoliosisjournal.com/supplements/10/S2.
